# Metformin Modulates Ferroptosis-Related and Antioxidant Gene Expression in Brown Adipose Tissue

**DOI:** 10.3390/ijms27177625

**Published:** 2026-08-25

**Authors:** Dong Soo Seo, Sungjun Park, Yusra Ahmad, Junhyeok Lee, Jeongwoo Yoo, Jaehyeon Kang, Wanjun Kim, Siwoo Lee, Huiyoung Kwon, Ho Jung Bae, Jin-A Park, Sungweon Ryoo, Younghoon Jang

**Affiliations:** 1 Department of AI-Integrated Biological Sciences, Changwon National University, Changwon 51140, Republic of Korea; dsseo@changwon.ac.kr (D.S.S.);; 2 Global Institute for Advanced Nanoscience & Technology (GIANT), Changwon National University, Changwon 51140, Republic of Korea; 3 School of Advanced Bioengineering, Glocal Advanced Institute of Science and Technology, Changwon National University, Changwon 51140, Republic of Korea; 4 MycoDx Co. Ltd., 7 Yongdong-ro 83 Beonan-gil, Uichang-gu, Changwon 51139, Republic of Korea

**Keywords:** metformin, brown adipocytes, BAT, ferroptosis, obesity

## Abstract

Metformin is a widely prescribed antidiabetic agent with pleiotropic effects extending beyond glycemic control, including anti-inflammatory and antioxidant actions. However, its impact on cytokine signaling and ferroptosis-related pathways in brown adipose tissue (BAT) remains poorly characterized. Here, we examined the transcriptional and metabolic responses to metformin in brown adipocytes and in a diet-induced obesity model. Differentiated brown adipocytes were treated with metformin and analyzed by RNA sequencing and RT-qPCR. C57BL/6 male mice fed a high-fat diet (HFD) were administered metformin to assess systemic metabolic parameters and BAT-specific responses, with validation by histology, RT-qPCR, and Western blot. Transcriptomic profiling identified differentially expressed genes enriched in cytokine–cytokine receptor interaction and ferroptosis-related KEGG pathways. Metformin modulated the expression of multiple inflammatory cytokine genes and upregulated antioxidant and ferroptosis defense-related genes, including the glutathione biosynthesis genes *Gclc* and *Gclm*, together with *Hmox1*, *Gpx4*, *Nfe2l2* (*Nrf2*), and *Slc7a11*. Among these, *Gpx4* showed the most consistent upregulation across mRNA and protein levels in BAT. In HFD-fed mice, metformin improved glucose tolerance and elevated the expression of antioxidant and ferroptosis-related genes in BAT, consistent with the in vitro findings. Together, these results indicate that metformin coordinately modulates antioxidant and ferroptosis defense-related gene expression in BAT, suggesting a tissue-protective transcriptional program under metabolic stress. Our findings identify candidate immunometabolic and ferroptosis-related targets of metformin in BAT and provide a basis for further mechanistic investigation of its tissue-specific actions beyond glycemic control.

## 1. Introduction

### 1.1. Metformin, Diabetes, Obesity, and Its Drug Repositioning in Other Diseases Such as Cancers

Metformin (1,1-dimethylbiguanide) is derived from Galega officinalis, commonly known as French lilac, a plant that has been used in traditional medicine for centuries [1]. It is the most widely prescribed oral medication used to treat type 2 diabetes (T2D) by reducing plasma glucose levels, both basal and postprandial. In addition to enhancing insulin sensitivity, it functions by decreasing glucose synthesis in the liver and intestinal glucose uptake [2]. Beyond its approved indication for type 2 diabetes, metformin has been investigated as an off-label option for obesity and NAFLD, reflecting its insulin-sensitizing and anti-inflammatory properties, although it is not approved for either indication [3]. It targets mitochondrial complex I and lysosomal presenilin enhancer protein 2 (PEN2) and regulates AMP levels and the 5′ adenosine monophosphate-activated protein kinase (AMPK) pathway [4]. Previous studies have shown that metformin decreases body weight through a GDF15-GFRAL axis, reduces food intake, and increases energy expenditure. Metformin’s tissue selectivity reflects its dependence on organic cation transporters for cellular entry, with *Gdf15* expression selectively increased in the small intestine, colon, and kidney, but not in skeletal muscle or adipose tissue [5]. Because uptake depends on transporter expression, metformin accumulates in the intestine, liver, and kidney, where tissue levels are higher than plasma levels [6]. Metformin, when combined with DNase I, helps inhibit the progression of obesity-induced pancreatic carcinogenesis by suppressing the formation of a fibroinflammatory microenvironment [7]. The underlying molecular mechanisms regulating metformin’s tissue-specific responses remain unclear [8]. Recent findings reveal that metformin-induced AMPK activation is closely correlated with its anti-tumor immune activity. First, metformin directly inhibits cancer proliferation by suppressing multiple anabolic pathways; second, in the TME, it restores the metabolic function of effector immune cells, thereby enhancing anti-tumor immunity [9]. In contrast, in metabolic tissues, metformin attenuates chronic low-grade inflammation by reducing the expression of proinflammatory cytokines and promoting an anti-inflammatory macrophage phenotype. In addition, AMPK activation by metformin facilitates p53 phosphorylation at serine 15 and 20 sites, which increases p53 activity and promotes an anti-inflammatory response [10]. The anti-cancer effect of metformin may also be facilitated through decreasing blood glucose levels and improving insulin sensitivity, leading to lower levels of insulin and insulin-like growth factor 1 (IGF-1), which can inhibit cancer growth [11]. In cancer cells, metformin reduces glucose oxidation, increases dependency on reductive glutamine metabolism, and lowers fatty acid oxidation (FAO) by suppressing the TCA cycle [12]. Metformin’s therapeutic effects extend far beyond its anti-glycemic role through a complex network of molecular mechanisms. These mechanisms explain metformin’s emerging role in treating various conditions, while highlighting the importance of characterizing tissue-specific responses to optimize therapeutic benefits.

### 1.2. Effects of Metformin in Adipose Tissues (WAT and BAT) (Understanding Metformin’s Role in Adipose Tissue Metabolism and Thermogenesis)

Recent studies have demonstrated that metformin administration in HFD-fed mice and T2D patients results in a reduction in adipose tissue. This reduction is attributed to increased uptake and utilization of VLDL-TG-derived fatty acids, leading to decreased tissue mass, lipid accumulation, and intracellular lipid content in brown adipose tissue (BAT) without altering mRNA and protein expression of UCP1 [13,14]. In metformin intervention studies, BAT demonstrated significant improvement, as indicated by enhanced proliferation and development of brown adipocytes in mice consuming a high-sugar diet [15]. In obese mouse models, metformin showed a therapeutic effect by upregulating genes involved in BAT differentiation [16]. In white adipose tissue (WAT), the accumulation of metformin enhances insulin-mediated glucose uptake, utilization, and metabolism. Moreover, other studies have demonstrated that metformin upregulates the expression of genes associated with BAT and beige adipose tissue, such as UCP1 and PRDM16, and facilitates mitochondrial biogenesis, thermogenesis, and fatty acid uptake [17]. Metformin exerts diverse effects on WAT, such as anti-inflammatory effects, metabolic improvements, and promoting adipose browning. These effects are primarily the result of AMPK signaling activation, altered macrophage polarization, and regulation of adipokine homeostasis. Collectively, these mechanisms are responsible for the antidiabetic and anti-obesity properties of metformin mediated via adipose tissue [18]. In obese T2D patients, metformin treatment reduces adipose tissue, thereby leading to weight loss. Moreover, metformin provides therapeutic benefits in improving hepatic steatosis [19]. In metformin-treated mice, there is a strong correlation between decreased adipose fibrosis and reduced fasting glucose levels. The improved metabolic function observed in mice receiving metformin treatment might be a secondary effect of reduced adiposity and inflammation [20]. Whether metformin also influences the oxidative and lipid peroxidation processes associated with thermogenesis in BAT remains unknown.

### 1.3. Effects of Metformin on Ferroptosis (Metformin’s Tissue-Specific Response in Ferroptosis Regulation)

Ferroptosis is a distinct type of non-apoptotic cell death resulting from interactions between iron, oxygen, and oxidizable phospholipids (PLs). The key feature differentiating ferroptosis from other cell death pathways is its hallmark accumulation of (phospho)lipid hydroperoxides [21]. Metformin has shown therapeutic potential in treating inflammation and lipid metabolic disorders, cancer, cardiovascular diseases, and kidney disorders, in addition to its well-known anti-diabetic effects [22,23]. Metformin exhibits anti-ferroptotic properties, which help improve hyperlipidemia-associated vascular calcification and protect pancreatic β cells from glucolipotoxicity-induced damage [24]. Metformin suppresses inflammatory pathways by reducing the expression of proinflammatory cytokines, including TNF-α, IL-6, and IL-1β [25]. Metformin strengthens the antioxidant defense mechanism in vascular calcification by promoting nuclear translocation of Nrf2 from the cytoplasm. Notably, enhanced Nrf2 protein expression can induce transcription of genes encoding antioxidant proteins and is known to control the function of various proteins associated with ferroptosis and lipid peroxidation [26]. Ferroptosis induction by metformin is facilitated by targeting the miR-324-3p/GPX4 axis or by lncRNA H19-regulated autophagy inhibition in breast cancer. In contrast, the combined use of metformin and sorafenib in hepatic cancer cells promotes ferroptosis through the p62-Keap1-Nrf2 pathway [27]. A previous study showed that metformin induces anti-ferroptosis in vascular smooth muscle cells, which may affect the pathological progression of vascular calcification associated with hyperlipidemia [28]. Recent research has revealed that metformin can inhibit tumor development by inducing ferroptosis through AMPK-independent pathways. Notably, metformin impairs the UFMylation of SLC7A11, an essential ferroptosis regulatory protein, causing its degradation [29]. Another study found that in lung cancer cell lines, metformin elevated ROS and iron ion concentrations while decreasing GPX4 and GSH levels; however, the addition of a ferroptosis inhibitor restored these depleted levels [30]. Collectively, these previous studies indicate that metformin’s ferroptotic response depends on the redox and lipid environment of the target tissue, but it remains unclear in the context of BAT, where the abundance of mitochondria and PUFA-rich membrane lipids may facilitate lipid peroxidation during thermogenesis. Using transcriptomic profiling of brown adipocytes together with in vivo analysis of chow and HFD-fed mice, our findings show a coordinated ferroptosis defense program involving *Gclc*, *Gclm*, *Slc7a11*, *Hmox1*, and *Gpx4*, along with preserved thermogenic capacity. In contrast to previous Nrf2-centered studies [26], we found that Gpx4 represents the most consistent effector across mRNA, protein, and tissue levels, and is upregulated even under HFD conditions where *Nfe2l2* expression remains unchanged.

## 2. Results

### 2.1. Transcriptome Analysis of Brown Adipocytes Under Metformin Treatment

**Figure 1:**
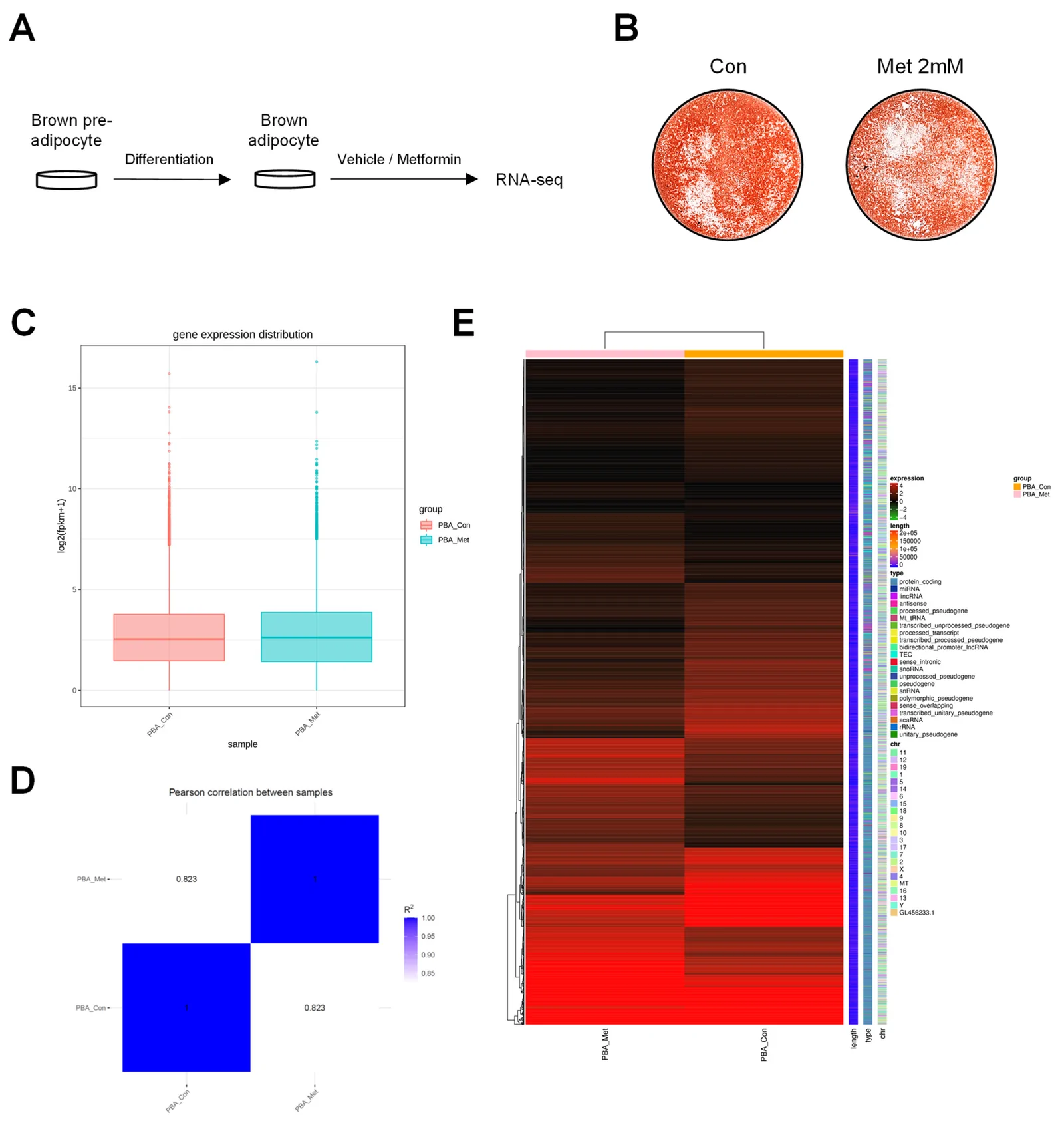
Metformin treatment induces transcriptional changes in differentiated brown adipocytes (**A**) Schematic illustration of the experimental workflow. Brown pre-adipocytes were differentiated into mature adipocytes and then treated with either vehicle control or metformin, followed by RNA sequencing analysis. (**B**) Oil Red O staining showing cellular lipid content in brown adipocytes treated with control or metformin (2 mM). (**C**) The box plot illustrates the distribution of gene expression levels (log2(fpkm + 1) between the control and metformin-treated groups, demonstrating equivalent overall expression ranges between experimental groups. (**D**) Pearson correlation analysis reveals a high correlation among biological replicates within each group (r^2^ = 0.823) and distinct clustering between control and metformin treatments, confirming sample quality and treatment-specific transcriptional changes. (**E**) Clustered heatmap of genes differentially expressed between the control and metformin-treated brown adipocytes. Genes are annotated with biotype information (protein coding, lncRNA, pseudogene, etc.) and length characteristics. The color gradient represents expression levels (red: elevated expression, black: reduced expression). Bottom labels denote treatment conditions (PBA_Met: metformin; PBA_Con: vehicle).

**Figure 2:**
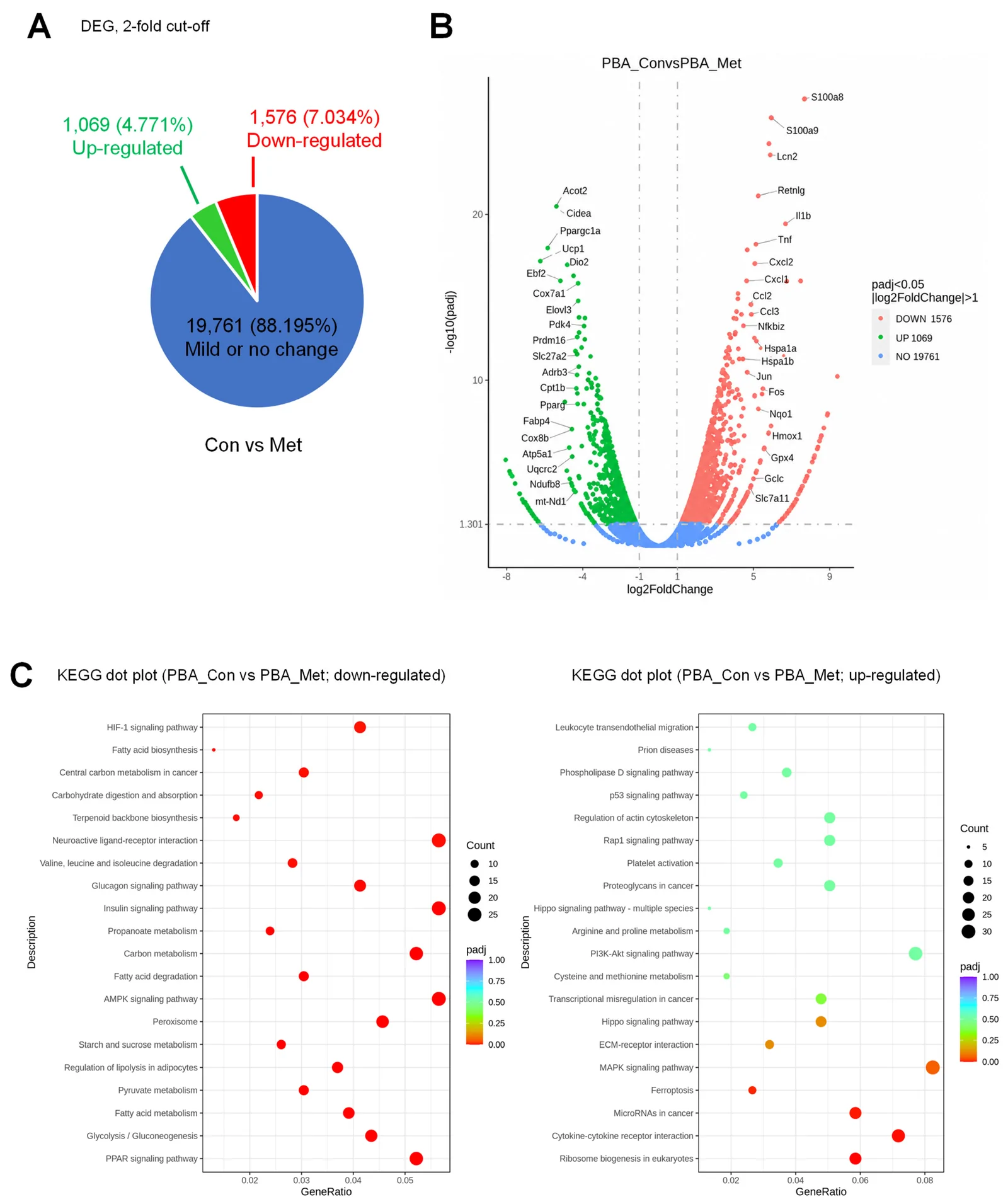
Differential gene expression and KEGG pathway analysis of metformin-treated brown adipocytes. (**A**) Pie chart depicting the distribution of differentially expressed genes (DEGs) between control and metformin-treated brown adipocytes with a 2-fold change threshold. A total of 2645 genes showed significant differential expression, with 1069 genes (4.772%) upregulated (green) and 1576 genes (7.034%) downregulated (red). The majority of genes (19761, 88.195%, blue) displayed minimal or no expression changes. (**B**) Volcano plot displaying the correlation between gene expression changes (log2FoldChange on the *x*-axis) and statistical significance (−log10(padj) on the *y*-axis) across all identified genes. Red dots represent significantly downregulated genes (1576 genes), green points show significantly upregulated genes (1069 genes), and blue points represent genes without significant changes (19,761 genes). Dashed vertical lines indicate ±1 log2 fold change threshold; the horizontal dashed line indicates an adjusted *p*-value < 0.05. (**C**) KEGG enrichment scatter plots showing downregulated genes (**left** panel) and upregulated genes (**right** panel). The gene ratio (*x*-axis) represents the proportion of differentially expressed genes within each pathway, and the dot size indicates the number of genes associated with that pathway. The color gradient represents adjusted *p*-values, where red and orange dots indicate greater significance of enrichment.

To investigate the effect of metformin on brown adipocytes’ gene expression, we differentiated brown pre-adipocytes into mature brown adipocytes and subsequently treated them with control or metformin (Figure 1A), followed by transcriptomic analysis to evaluate the transcriptional responses mediated by metformin treatment in mature brown adipocytes. Oil Red O staining revealed lipid accumulation in metformin (2 mM)-treated cells and vehicle controls (Figure 1B). RNA sequencing showed distinct clustering between treatment groups, where metformin substantially modified the transcriptomic landscape (Figure 1C). Pearson correlation analysis confirmed strong concordance among biological replicates within each group, with distinct separation between control and metformin-treated groups (Figure 1D). The heatmap revealed alterations in gene expression patterns, indicating that metformin modulates adipogenic transcriptional pathways (Figure 1E). Using a 2-fold change cutoff, we identified that 1069 (4.8%) genes were upregulated and 1576 (7.1%) genes were downregulated in the metformin-treated group (Figure 2A). The volcano plot displayed the extent and statistical significance of gene expression alterations. The plot clearly distinguishes between upregulated and downregulated genes, confirming that metformin induces targeted transcriptional changes in brown adipocytes and facilitates further analysis of ferroptosis pathways (Figure 2B). KEGG pathway analysis indicated that metformin treatment resulted in the upregulation of cytokine-cytokine receptor interaction and stress response pathways, with targeted gene expression analysis demonstrating increased expression of key ferroptosis defense genes (Figure 2C). These results demonstrate that metformin significantly modifies the gene expression patterns of brown adipocytes, with substantial upregulation of anti-ferroptosis genes and cytokine signaling pathways. These molecular changes offer mechanistic insights into metformin’s protective effects and highlight potential therapeutic targets for metabolic intervention.

### 2.2. Metformin Alters Inflammatory Cytokine Gene Expression in Brown Adipocytes

**Figure 3:**
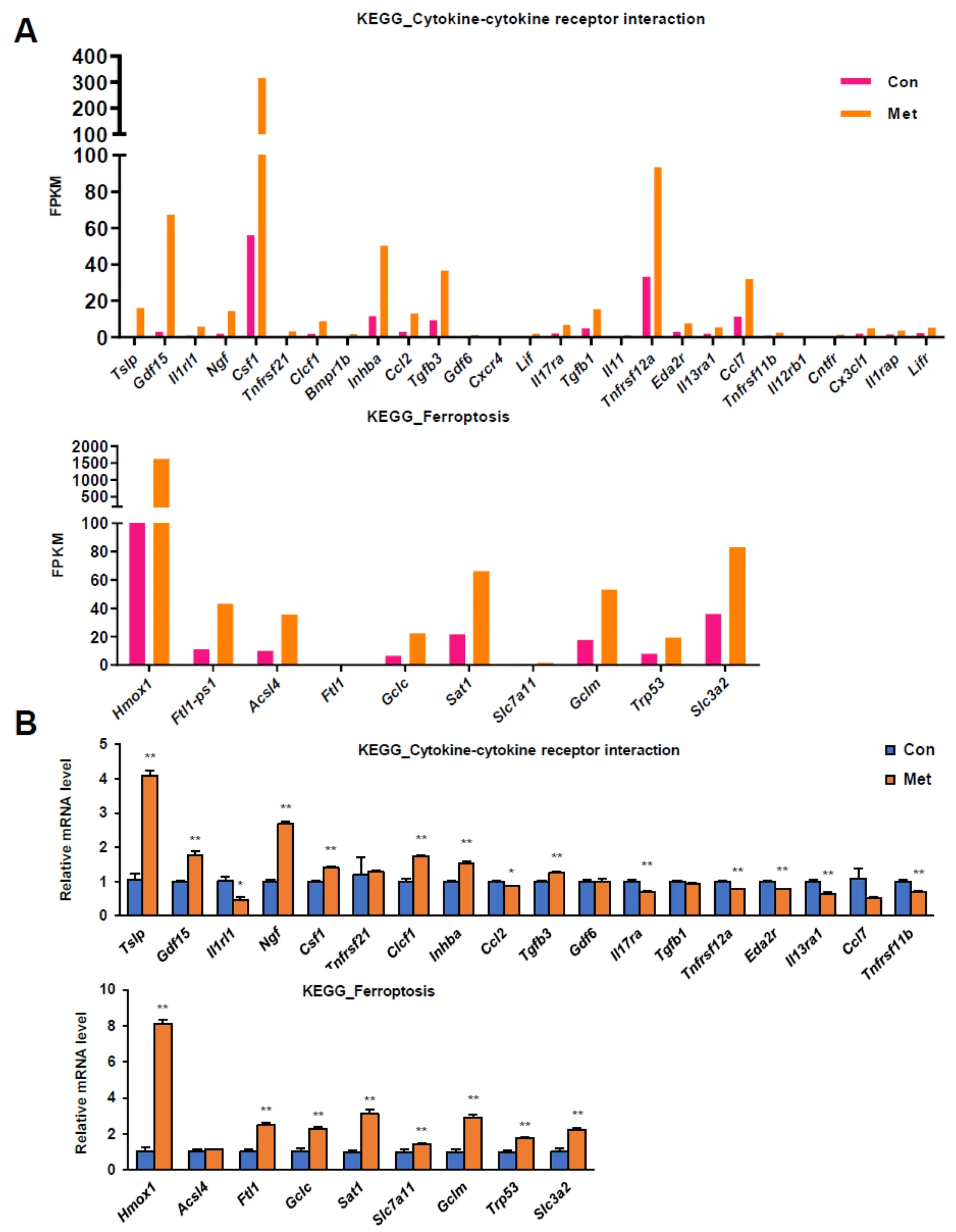
RNA-seq and RT-qPCR analysis indicate enrichment of cytokine signaling and ferroptosis pathways. (**A**) KEGG pathway enrichment analysis reveals FPKM values for genes involved in the cytokine–cytokine receptor interaction and ferroptosis pathways. In the cytokine–cytokine receptor interaction pathway, metformin treatment increased the expression of multiple genes, with *Csf1*, *Gdf15*, and *Tnfrsf1a* showing the highest expression levels. Ferroptosis-related genes also exhibited increased expression in the metformin-treated group, particularly *Hmox1* and *Slc3a2*. (**B**) RT-qPCR validation confirmed the expression pattern observed in the RNA-seq analysis. Several cytokine-related genes show upregulation in the metformin group. Ferroptosis-related genes were also elevated in the metformin group, particularly Hmox1, which shows the highest fold change (~8-fold), followed by other key ferroptosis regulators including *Gclc*, *Sat1*, *Slc7a11*, and *Slc3a2*. Data are presented as mean ± SEM (*n* = 3 per group) * *p* < 0.05, ** *p* < 0.01.

KEGG pathway enrichment analysis demonstrated enhanced activity of the cytokine–cytokine receptor interaction and ferroptosis pathways in the metformin-treated group compared to the control. The cytokine–cytokine receptor interaction pathway showed the highest enrichment scores, with multiple inflammatory factors significantly upregulated. The ferroptosis pathway also suggested activation of ferroptosis-related genes in response to metformin (Figure 3A). qRT-PCR analysis validated these findings at the transcriptional level in both pathways. Metformin treatment resulted in a substantial upregulation of key anti-ferroptosis factors, including *Hmox1*, which exhibited the most dramatic increase, followed by upregulation of *Gclc*, *Sat1*, *Slc7a11*, *Gclm*, and *Slc3a2*. This confirms that metformin regulates the expression of genes involved in ferroptosis resistance, supporting our hypothesis that metformin provides protective effects against ferroptosis by enhancing anti-ferroptotic pathways (Figure 3B). The pathway enrichment analysis revealed that metformin activates a coordinated cellular protective response in brown adipocytes while upregulating anti-ferroptotic factors and modulating inflammatory signaling networks. These findings are consistent with enhanced antioxidant capacity in brown adipocytes during cellular stress.

### 2.3. Effects of Metformin on Systemic Metabolism in Chow-Fed Mice

**Figure 4:**
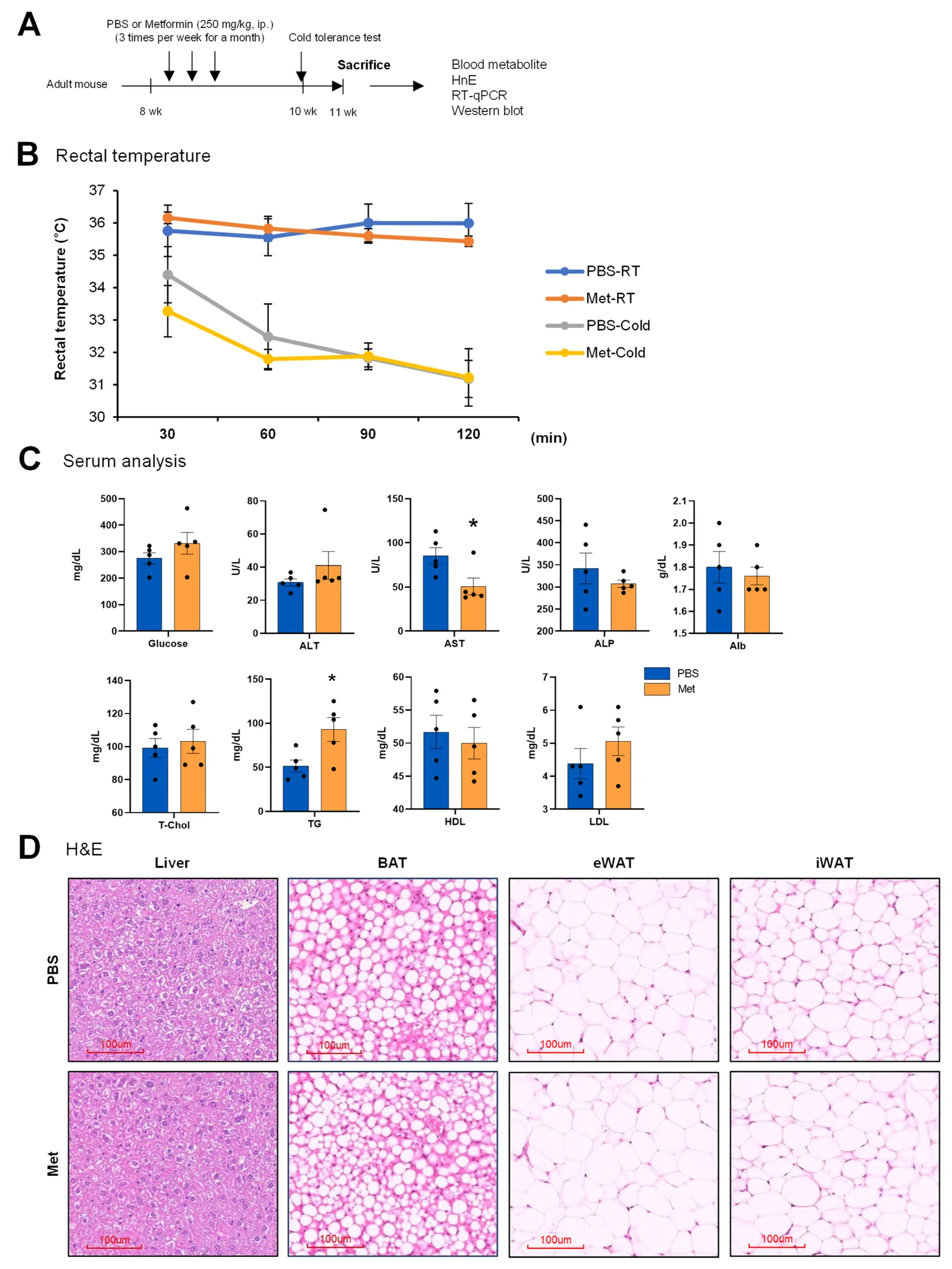
In vivo metabolic and histological characterization of metformin-treated lean mice. (**A**) Schematic diagram of the experimental protocol. Adult mice received PBS and metformin (250 mg/kg, 3 times/week) for a month, and at 11 weeks of age, the mice were sacrificed to collect blood and tissues for further metabolic analysis. (**B**) Rectal temperature was measured during cold exposure. Mice were monitored for over 120 min at 4 °C. Mice treated with metformin and PBS showed comparable thermoregulatory responses during cold exposure (*n* = 6 per group). (**C**) Serum biochemical analysis showed a trend toward elevated glucose levels in the metformin group. The liver function marker AST was significantly reduced in the metformin group, while ALT showed a modest increase. Lipid profile analysis demonstrated a significant increase in triglycerides (TG) in the metformin group, while T-Chol, HDL, and LDL showed no significant differences between groups. Data are presented as mean ± SEM (*n* = 5 per group) * *p* < 0.05. (**D**) Representative H&E staining images of the liver, BAT, eWAT, and iWAT in both PBS and metformin groups show no apparent pathological changes. Scale bars = 100 μm.

To study the metabolic effects of metformin treatment, we treated mice with metformin (250 mg/kg) three times per week for a month (Figure 4A). At 10 weeks, we performed a cold tolerance test (CTT) and monitored rectal temperature, which revealed that cold-exposed metformin-treated and PBS mice showed comparable temperature profiles (Figure 4B). At 11 weeks, the mice were sacrificed and tissues and blood were collected. Serum analysis was performed, which revealed no significant alterations in metabolic parameters following metformin administration, including glucose levels, lipid profiles (triglycerides and cholesterol), and hepatic enzyme levels (Figure 4C). Histological examination revealed no overt differences in lipid droplet morphology or adipocyte appearance in BAT, eWAT, or iWAT between the metformin- and PBS-treated groups (Figure 4D). These metabolic data indicate that metformin treatment resulted in no significant alterations in thermogenic function, adipose tissue structure, and circulating metabolic markers.

### 2.4. Metformin Modulates Ferroptosis-Related Gene and Protein Expression in BAT

**Figure 5:**
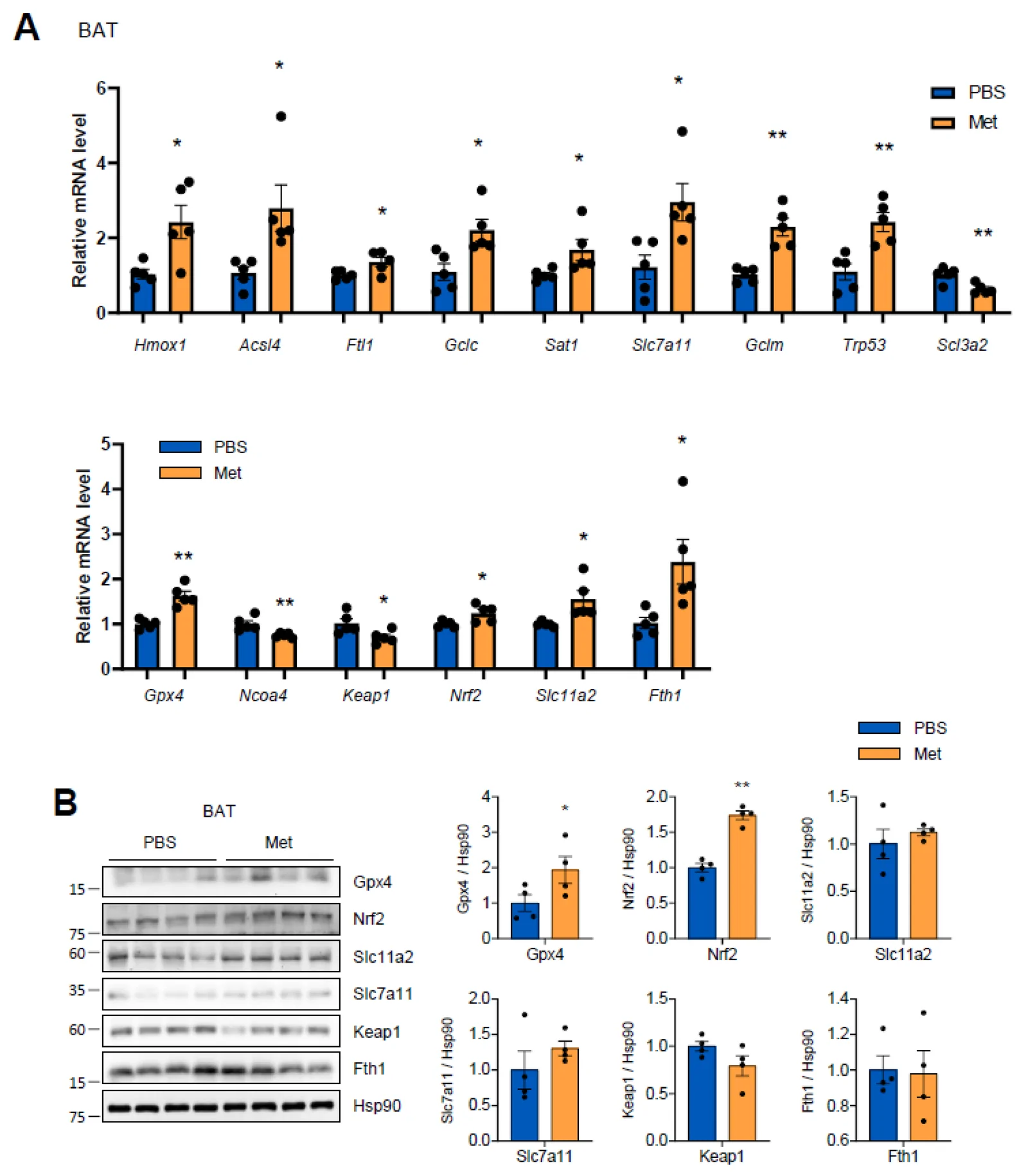
Gene and protein expression analysis of ferroptosis pathway components in metformin-treated BAT. (**A**) RT-qPCR analysis of key ferroptosis-related genes in both the control PBS and metformin-treated groups. Multiple ferroptosis-related genes, including *Hmox1*, *Acsl4*, *Ftl1*, *Gclc*, *Sat1*, *Slc7a11*, *Gclm*, and *Trp53*, showed upregulation in the metformin group. Additional mRNA expression profiling revealed changes in thermogenic and mitochondrial genes in BAT. The expression of *Gpx4*, *Nrf2*, *Slc11a2*, and *Fth1* was increased in the metformin group. These findings indicate that metformin treatment regulates ferroptosis-related pathways and oxidative stress responses in BAT. Data are presented as mean ± SEM (*n* = 5 per group); * *p* < 0.05, ** *p* < 0.01. (**B**) Western blot analysis and quantification of key ferroptosis regulators in BAT. Hsp90 was used as a loading control. Metformin treatment significantly increased the expression of Gpx4, Nrf2, and Slc7a11 proteins. Keap1 showed a non-significant decreasing trend, while Slc11a2 and Fth1 were unchanged. These results validate that metformin modulates key antioxidant and ferroptosis regulatory proteins in BAT at the protein level. Data are presented as mean ± SEM (*n* = 4 per group); * *p* < 0.05, ** *p* < 0.01.

Our RNA-seq data showed that metformin treatment resulted in significant upregulation of key anti-ferroptosis genes (Figure 3A). A recent study also demonstrated that metformin treatment reduced ferroptosis by upregulating anti-ferroptotic factors (Gpx4, GSH, SLC7A11) and decreasing lipid peroxidation. Additionally, metformin improved antioxidant activity by promoting Nrf2 nuclear transport, which enhances the transcription of protective genes and regulation of the ferroptosis pathway [26]. Consistent with these findings, the literature, and our RNA-seq data, our qRT-PCR analysis extensively validated that metformin treatment induced significant transcriptional upregulation of anti-ferroptosis genes, including genes involved in iron storage (*Ftl1*, *Fth1*), glutathione synthesis (*Gclc*, *Gclm*), cystine transport (*Slc7a11*), and antioxidant defense (*Gpx4*, *Nrf2*, *Slc11a2*). This coordinated upregulation indicates that metformin exerts its protective effects through systematic enhancement of cellular anti-ferroptotic pathways (Figure 5A). To further confirm the transcriptional findings, we performed Western blot analysis to measure the protein expression of key ferroptosis regulators in BAT. Consistent with the mRNA results, metformin administration resulted in upregulation of anti-ferroptosis protein levels, including Gpx4, Nrf2, and Slc7a11, compared with PBS controls. Keap1 protein levels showed a decreasing trend in the metformin-treated group that did not reach statistical significance, while Slc11a2 and Fth1 levels were unchanged (Figure 5B). These findings suggest that metformin upregulates ferroptosis defense factors at both transcriptional and translational levels, supporting its potential role in mitigating ferroptotic cell death through the enhancement of key cellular defense mechanisms.

### 2.5. Metabolic Effects of Metformin in Diet-Induced Obese Mice

**Figure 6:**
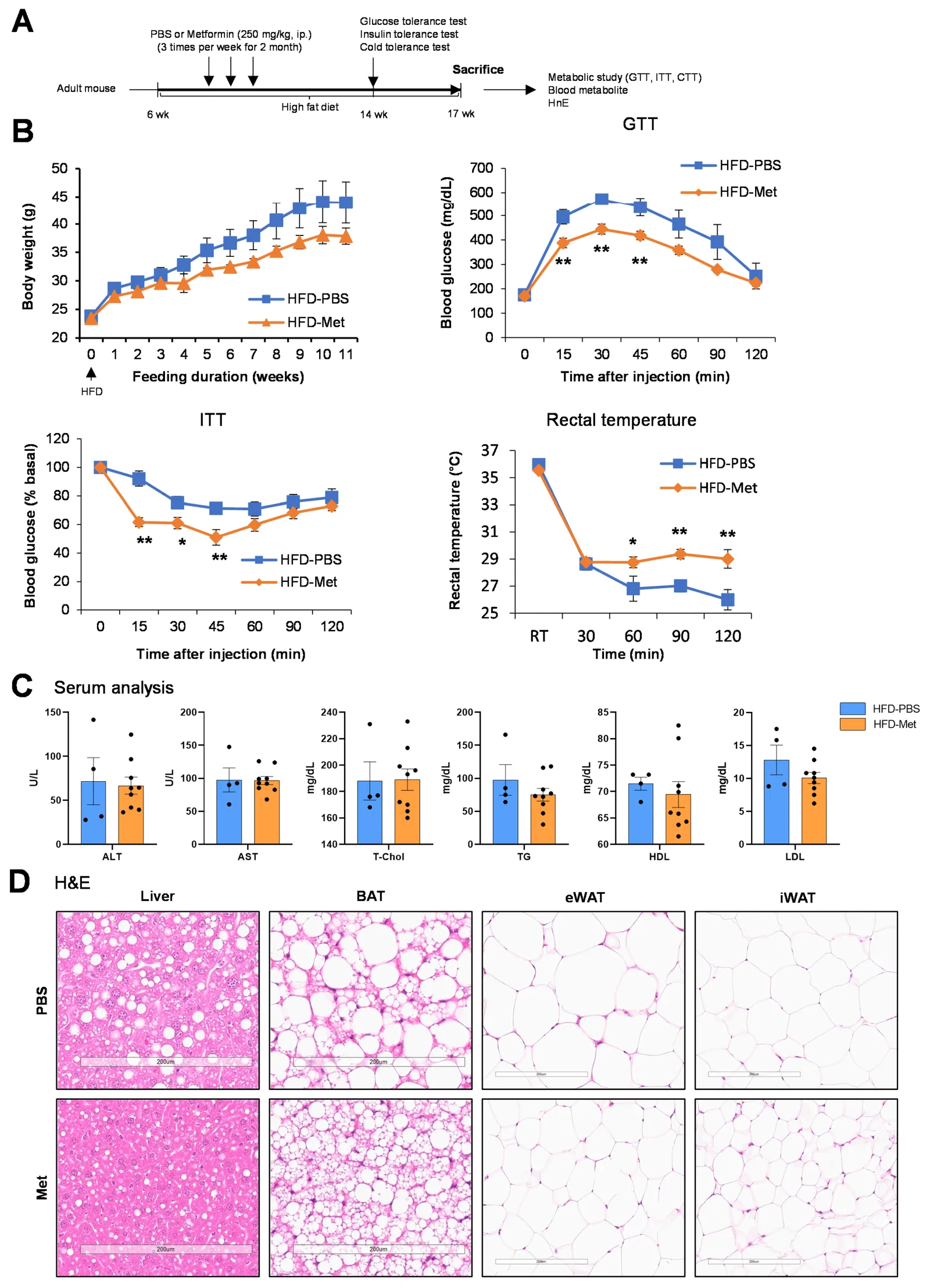
Metabolic effects of metformin treatment in a diet-induced obesity model. (**A**) Schematic of experimental design. Adult mice were fed HFD and received either PBS or metformin (3 times/week) for 2 months. GTT, ITT, and CTT were performed at 14 weeks of age. The mice were sacrificed at 17 weeks of age, and blood and tissues were collected. (**B**) Metabolic phenotyping of HFD-fed mice. Body weight showed comparable weight gain in both groups over the 11-week feeding period. GTT revealed significantly improved glucose clearance in the metformin group. ITT demonstrated enhanced insulin sensitivity in the HFD-fed metformin group. In contrast, CTT revealed a significant difference in rectal temperature between the two groups during 120 min of cold exposure at 4 °C. Data are presented as mean ± SEM (*n* = 6 for HFD-PBS and *n* = 9 for HFD-Met); * *p* < 0.05, ** *p* < 0.01. (**C**) Serum analysis revealed no notable differences between groups in liver function markers (ALT, AST) or lipid profiles (T-Chol, TG, HDL, LDL), indicating that metformin treatment did not alter liver function or circulating lipid levels in the context of HFD feeding. Data are presented as mean ± SEM (*n* = 6 for HFD-PBS and *n* = 9 for HFD-Met). (**D**) Representative H&E staining images of the liver, BAT, eWAT, and iWAT in both PBS and metformin groups show apparent pathological changes. Scale bars = 200 μm.

To demonstrate the metabolic effect of metformin under conditions of dietary stress, adult mice were fed a high-fat diet (HFD) and treated with PBS and metformin (250 mg/kg) three times per week for 2 months. At 14 weeks, we performed a glucose tolerance test (GTT) to assess glucose homeostasis, followed by an insulin tolerance test (ITT) and a cold tolerance test (CTT). At 17 weeks, mice were anesthetized using isoflurane and sacrificed. Blood and tissues were collected for further metabolic analysis to evaluate the long-term effect of metformin treatment in the context of HFD feeding (Figure 6A). Throughout the 11-week HFD feeding period, metformin treatment significantly reduced weight gain in HFD-fed mice compared to controls, with a clear separation between groups after week 6. Metformin treatment significantly improved glucose homeostasis, as evidenced by the GTT. The HFD-Met group showed markedly lower blood glucose levels at 15, 30, 45, and 60 min post-glucose injection compared to HFD-PBS (** *p* < 0.01), indicating enhanced glucose tolerance under HFD conditions. Similarly, the ITT revealed enhanced insulin sensitivity in the HFD-Met group, with improved glucose clearance at 15, 30, and 45 minutes post-insulin injection compared to HFD-PBS controls (* *p* < 0.05, ** *p* < 0.01), reflecting better insulin responsiveness. Consistent with the improvements in glucose metabolism, the CTT revealed that the HFD-Met group maintained a significantly higher rectal temperature than the HFD-PBS group at 60, 90, and 120 min during cold exposure, indicating improved thermoregulatory capacity following metformin treatment (Figure 6B). Serum analysis revealed no significant differences in liver function markers (ALT and AST) and lipid parameters (total cholesterol, triglycerides, HDL, LDL) between the HFD-Met and HFD-PBS groups. These results indicated that metformin treatment did not significantly impact liver function and lipid metabolism under these experimental conditions (Figure 6C). H&E staining revealed marked hepatic steatosis in the PBS group, evidenced by increased lipid droplet accumulation, which was significantly reduced in the Met-treated group. In the BAT group, the Met group exhibited smaller lipid droplets compared to the PBS group. Similarly, adipocytes in both eWAT and iWAT of PBS-treated mice displayed marked hypertrophic expansion, which was reversed by metformin treatment (Figure 6D). Because the cytokine–cytokine receptor interaction pathway was significantly enriched in the PBA RNA-seq analysis, cytokine-related genes showing differential expression in PBA, including *Tslp*, *Gdf15*, *Ngf*, *Csf1*, *Inhba*, and *Tgfb3*, were further examined by qPCR in the BAT of HFD-fed mice. However, none of these genes showed significant differences in vivo (Supplementary Figure S1).

### 2.6. Metformin Upregulates Antioxidant and Ferroptosis-Related Gene Expression in the BAT of HFD-Fed Mice

**Figure 7:**
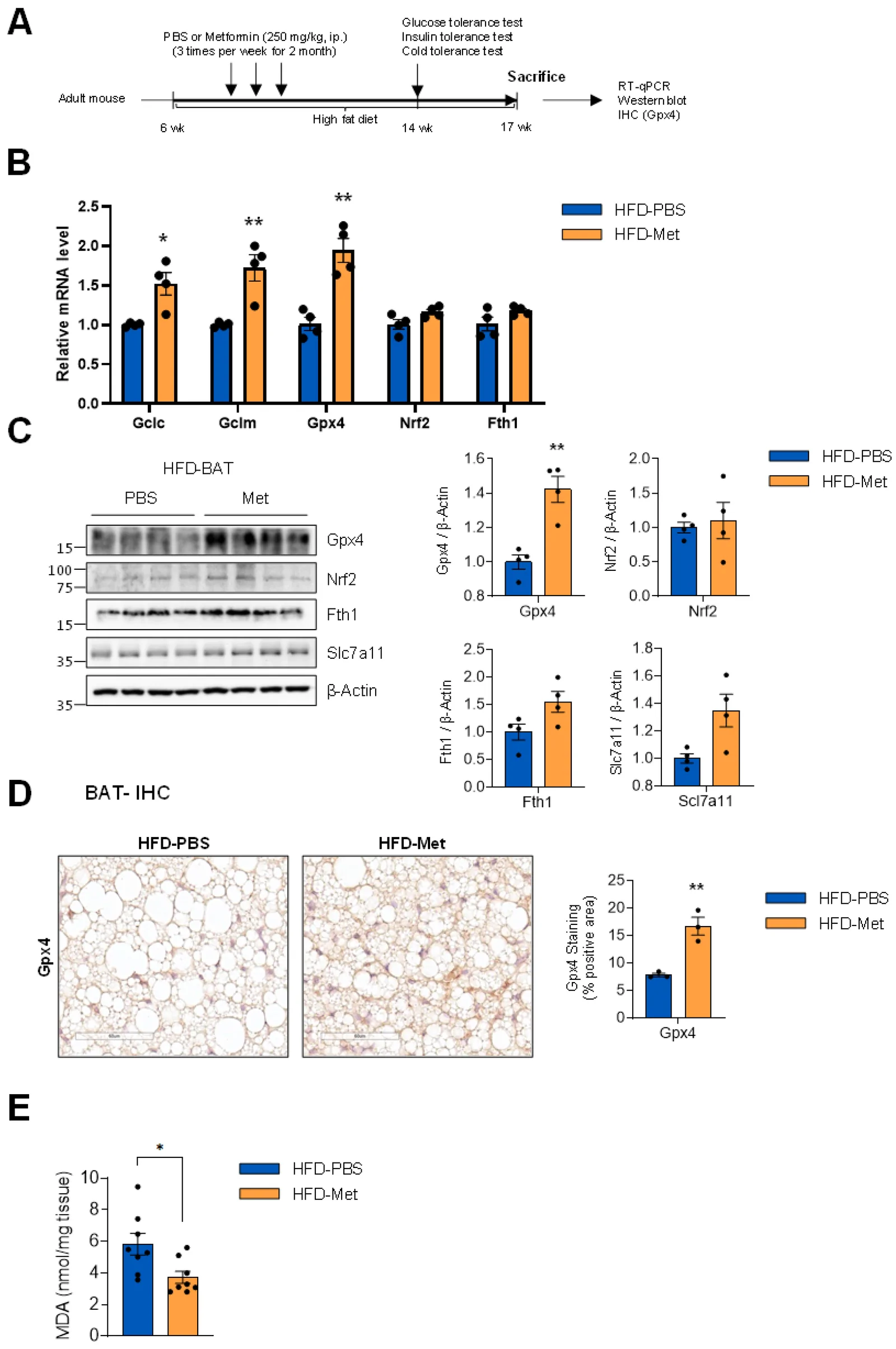
Metformin upregulates Gpx4 and antioxidant pathway components in the BAT of HFD-fed mice. (**A**) Overview of the experimental design. Adult, 6-week-old mice were fed an HFD and treated with either PBS or metformin (250 mg/kg, 3 times per week) for 2 months. GTT, ITT, and CTT were performed at 14 weeks, and then the mice were sacrificed at 17 weeks, and BAT tissues were collected for further experiments. (**B**) Relative mRNA levels of *Gclc*, *Gclm*, *Gpx4*, *Nrf2*, and *Fth1* in BAT were determined by RT-qPCR analysis. Data are presented as mean ± SEM (*n* = 4 per group); * *p* < 0.05, ** *p* < 0.01. (**C**) Representative Western blot images and densitometric quantification of Gpx4, Nrf2, Fth1, and Slc7a11 protein expression in BAT from HFD-PBS and HFD-metformin groups. β-Actin was used as a loading control. Data are presented as mean ± SEM (*n* = 4 per group); ** *p* < 0.01. (**D**) IHC images of Gpx4 staining in BAT sections from HFD-PBS and HFD-metformin mice. Scale bar = 60 µm. Quantification of Gpx4-positive staining area is expressed as a percentage of total tissue area. Quantitative data are presented as mean ± SEM; ** *p* < 0.01. (**E**) MDA content in BAT from HFD-PBS and HFD-metformin mice, expressed as nmol/mg tissue. Data are presented as mean ± SEM (*n* = 8 per group); * *p* < 0.05.

Since metformin has been implicated in cellular stress regulation, we investigated whether it modulates ferroptosis defense and oxidative stress pathways in BAT under HFD conditions. Adult 6-week-old mice were treated with either PBS or metformin (250 mg/kg, 3 times per week) for 2 months and given HFD. At 14 weeks, GTT, ITT, and CTT were performed, and at 17 weeks, the mice were sacrificed, and tissues were collected for further downstream analysis (Figure 7A). HFD mice treated with metformin showed a significant increase in the mRNA levels of *Gclc*, *Gclm*, and *Gpx4* in BAT compared to the HFD-PBS group. In contrast, the *Nrf2* and *Fth1* levels remained unchanged in either group (Figure 7B). Consistent with the qPCR results, the Western blot analysis also showed the upregulated protein expression of Gpx4 in the HFD-Metformin group relative to the control. Collectively, these data suggest that Gpx4 is the most consistently upregulated antioxidant factor in BAT (Figure 7C). To further validate Gpx4 protein expression at the tissue level, IHC staining was performed on BAT sections of both groups, revealing markedly denser and more widespread Gpx4-positive staining throughout the adipocyte-rich tissue of the metformin-treated group (Figure 7D). To evaluate the functional impact of ferroptosis defense gene induction on lipid peroxidation, we measured malondialdehyde (MDA) content in BAT. MDA levels were significantly reduced in the metformin-treated group compared with PBS controls, consistent with the observed upregulation of *Gclc*, *Gclm*, and *Gpx4* (Figure 7E). Taken together, these findings suggest that metformin enhances the expression of Gpx4 in BAT at both the biochemical and tissue levels, pointing to its potential involvement in modulating ferroptosis-related defense mechanisms in the context of HFD-induced metabolic stress.

## 3. Discussion

This study provides novel insights into the molecular mechanisms by which metformin influences brown adipocyte metabolism and cellular protection. Metformin enhances both proliferative and differentiation markers in brown adipocytes, likely through an AMPK-dependent mechanism [31]. Our comprehensive transcriptomic analysis revealed that metformin treatment alters the gene expression patterns in brown adipocytes, particularly enhancing anti-ferroptosis pathways and inflammatory mediator responses. The identification of upregulated and downregulated genes demonstrates metformin’s potential to exert transcriptional modifications, suggesting its broad therapeutic effects beyond traditional metabolic interventions. Beyond its anti-glycemic properties, metformin demonstrates diverse biological effects through various mechanisms that directly and indirectly target multiple pathways associated with diabetic complications, especially those regulating inflammation, thrombosis, and oxidative stress [32]. Our results strongly support the hypothesis that metformin exerts protective effects against ferroptosis by upregulating crucial ferroptosis-suppressing genes. The upregulation of specific genes such as *Hmox1*, *Gclc*, *Slc7a11*, *Gclm*, and *Gpx4* indicates that metformin induces several pathways associated with ferroptosis resistance. This is noteworthy because ferroptosis has been identified as a primary cell death mechanism in metabolic pathophysiology and aging processes. Ferroptosis is a specialized cell death process characterized by iron-dependent lipid peroxidation, which distinguishes it from apoptosis, necrosis, and autophagy through its unique morphological, biochemical, and genetic features. This process is initiated by the inactivation of glutathione (GSH)-dependent cellular antioxidant defenses, resulting in the accumulation of toxic lipid ROS [33,34]. In our study, enhanced expression of glutathione synthesis genes (*Gclc*, *Gclm*) and the cystine-glutamate antiporter (Slc7a11) indicates that metformin strengthens cellular antioxidant defenses by maintaining adequate glutathione availability.

Additionally, the increased expression of Gpx4, the key enzyme responsible for decreasing lipid hydroperoxides, protects against ferroptotic cell death. Ferroptosis-mediated cell death occurs through the fatal combination of iron toxicity, antioxidant depletion resulting from GPX4 disruption, and membrane destruction caused by polyunsaturated phospholipid peroxidation [35]. The correlation between gene expression and protein level modifications indicates that these transcriptional changes are reflected at the protein level. Notably, the expression of antioxidant and ferroptosis defense genes was upregulated in BAT in chow-fed mice in the absence of significant systemic metabolic changes. This finding suggests that the response occurs independently of improved glycemic control and may reflect a direct action of metformin within the tissue. These results are consistent with recent studies demonstrating metformin’s protective role against ferroptosis through enhanced expression of anti-ferroptotic genes and increased antioxidant function. Metformin reduced ferroptosis by increasing the expression of anti-ferroptosis factors (Gpx4, GSH, and SLC7A11), alongside reduced lipid peroxidation [26].

The elevation of Nrf2 levels and its downstream targets serve as a key pathway through which metformin mediates cellular defense. Nrf2 acts as a pivotal regulator of the antioxidant response, and its enhancement is likely to upregulate multiple anti-ferroptotic genes observed in our study. These results support previous findings showing metformin’s ability to upregulate Nrf2 signaling via AMPK-dependent processes. Metformin can indirectly regulate Nrf2 signaling through AMPK-dependent serine 550 phosphorylation of Nrf2, facilitating enhanced interactions with key Nrf2 activators [36]. However, our study notably demonstrates this mechanism specifically in brown adipocytes within the context of ferroptosis protection. The nuclear translocation of Nrf2 enhances the expression of protective genes, especially those regulating glutathione production and iron metabolism, which are essential for ferroptosis resistance. Nrf2 regulates the ferroptosis pathway either directly or indirectly by modulating diverse cellular components, including GPX4 protein levels, intracellular free iron concentrations, mitochondrial functionality, and NADPH regeneration, among other factors [37]. The upregulation of cytokine-cytokine receptor interaction pathways represents a notable finding that warrants further investigation.

Although inflammation is commonly considered harmful, the metformin-mediated inflammatory response may unexpectedly act as a protective regulatory mechanism, improving the cellular ability to resist stressful conditions. This controlled inflammatory response could explain metformin’s beneficial effects under stress conditions, where low-level cellular stress enhances overall resistance and survival. Our findings have important implications for understanding metformin’s therapeutic effects beyond glycemic control. Metformin has emerged as the most studied hypoglycemic drug, showing remarkable therapeutic effects beyond glucose control through systematic research. Its therapeutic properties involve targeting different stages of carcinogenesis, aiding in weight reduction, and treating thyroid diseases, among other clinical conditions [38]. The substantial anti-ferroptosis effects indicate that metformin could protect against age-related metabolic disorders, where ferroptosis contributes to cellular damage. As previously described, aging is influenced by oxidative stress, and ferroptosis may contribute to this process, ultimately leading to cell death [39]. The identification of targeted ferroptosis-related markers (Hmox1, Gpx4, Slc7a11) that respond to metformin treatment could inform the development of personalized therapeutic plans and clinical monitoring strategies for patients receiving metformin. These biomarkers may also serve as drug targets for developing novel therapeutics that mimic metformin’s protective effects. The metformin dose used in this study (250 mg/kg, i.p., three times weekly) corresponds to a human-equivalent dose of approximately 20.3 mg/kg based on body surface area-based conversion, equivalent to approximately 1.2–1.4 g/day for a 60–70 kg adult [40]. This dose is comparable to commonly used clinical doses of metformin (1000–2000 mg/day) [41], indicating that the responses described here occur within a clinically relevant dose range. Furthermore, whether the ferroptosis-associated antioxidant responses described here contribute to the metabolic benefits observed in metformin-treated patients remains to be determined.

This study has several limitations. Although our findings demonstrate an association between metformin treatment and enhanced ferroptosis defense, as shown by increased *Gpx4* expression, the relationship among AMPK activation, Nrf2 signaling, and Gpx4 upregulation was not experimentally established. AMPK activity was not directly assessed, and pathway-specific inhibitors or gene silencing approaches were not used. Additionally, this study was conducted only in male mice. Considering the recognized sex-specific differences in BAT activity and metformin responsiveness, whether a similar antioxidant and ferroptosis defense response occurs in females remains unknown and requires further investigation. Furthermore, our findings do not demonstrate that the systemic metabolic improvement seen in HFD-fed mice is due to metformin action in BAT. Since metformin targets several tissues, including the liver, skeletal muscle, and intestine, establishing a BAT-specific contribution would require surgical ablation or denervation models. Related to this, metformin concentrations in plasma and BAT were not measured, so the internal exposure resulting from our dosing protocol, and its comparability with clinical exposure, remain undetermined. Similarly, although *Gpx4* was the most consistently upregulated factor in BAT at the transcript, protein, and tissue levels, its functional involvement in the observed responses was not directly tested. Confirming the mediating role of *Gpx4* would require BAT-specific *Gpx4* knockout or knockdown models under HFD conditions.

## 4. Materials and Methods

### 4.1. Antibodies and Chemicals

Anti-Fth1 (#4393), anti-Nrf2 (#12721), anti-Slc7a11 (#12691), anti-Keap1 (#8047), anti-Gpx4 (#52455 and #59735), anti-Slc11a2 (#15083), anti-Hsp90 (#4874), and beta-Actin (#4967) were obtained from Cell Signaling Technology (Danvers, MA, USA).

### 4.2. Mice and Diets

Eight-week-old wild-type male mice were purchased from SAMTACO (Gyeonggi, Republic of Korea). Mice were housed in a controlled environment (12-h light/dark cycle at 24 °C) with unlimited access to a normal chow diet (NCD) (Altromin International, 1314 IRR) before experimental procedures. To establish the diet-induced obesity model, the mice were given a high-fat diet (HFD) consisting of 20% carbohydrates, 20% protein, and 60% fat (Research Diets, D12492) for 8 weeks. For treatment, the mice were randomly assigned and intraperitoneally injected with metformin (D150959, sigma, 250 mg/kg) or PBS. All mouse-related experiments were conducted in accordance with the standards set by the Institutional Animal Care and Use Committee at Changwon National University and in compliance with institutional ethical requirements (approval number: 2022-03, Date of approval: 20 May 2022).

### 4.3. Cold Tolerance Assay

To perform a cold tolerance assay, the mice were placed individually in a cold room maintained at 4 °C. The body temperature was recorded using a rectal thermometer (Testo 925, Type K Thermocouple, Testo SE & Co. KGaA, Titisee-Neustadt, Germany) immediately after exposure to a cold room (at 0 min) and then at 30-min intervals for 2 h (at 30, 60, 90, and 120 min post-exposure). If the core body temperature of the mice dropped by more than 10 °C from baseline values, they were humanely euthanized in accordance with IACUC-approved protocols, and the final recorded values were used in data analysis.

### 4.4. Primary Preadipocyte Culture and Adipogenesis

Primary brown preadipocytes were harvested from the interscapular BAT of newborn wild-type mouse pups. For adipogenic differentiation, preadipocytes were cultured in growth medium (DMEM supplemented with 10% FBS) for 3–4 days before induction. Differentiation was initiated using an induction cocktail containing 0.02 μM insulin, 1 nM T3, 0.5 mM IBMX, 2 μM dexamethasone, and 0.125 mM indomethacin for 2 days. After differentiation was complete (day 8), fully differentiated brown adipocytes were treated with 2 mM metformin for 24 h.

### 4.5. RNA-Seq and Data Analysis

PBA was isolated and differentiated in vitro, followed by treatment with 2 mM metformin for 24 h. RNA-seq analysis was conducted according to previously established protocols [4,42] on an Illumina NovaSeq 6000 platform (Illumina, San Diego, CA, USA), following the manufacturer’s instructions. RNA extraction was carried out using an RNA purification kit (Qiagen, Hilden, Germany) according to the manufacturer’s protocol. For each experimental group, RNA samples from three independent biological samples were pooled into a single sample prior to library preparation and sequencing. Thus, one pooled RNA sample per group was subjected to RNA-seq (*n* = 1 per group). The control and metformin-treated samples generated 47.19 million and 55.24 million clean reads, respectively. The Q30 scores were 94.28% and 93.94%, and the GC contents were 49.91% and 50.36%, respectively. The sequencing error rates were 0.02% and 0.03%, respectively. mRNA sequencing, along with data analysis, was performed as previously described [4]. FastQC software (v0.12.1) was used to evaluate the raw sequencing data. Read mapping to the mouse mm9 genome assembly was performed using Hisat2 v and quantified with featureCounts v. 1.5.0-p3 for calculating reads per gene. FPKM values were determined based on the length and number of mapped reads, as sourced from the table. The FPKM clustering heatmaps were generated using log2(FPKM + 1) processed expression data. Gene expression intensity is displayed through color gradients, where red indicates high expression levels and blue represents low expression levels. The color gradient from red to blue illustrates log2(FPKM + 1) values from high to low. Gene FPKM values were organized using an established hierarchical clustering method, along with Z-score standardization of rows. Similar expression patterns of genes and samples were clustered together in a heatmap. Cell colors represent the normalized expression values obtained from row standardization, rather than the original gene expression values. Therefore, heatmap colors are only interpretable horizontally between samples for each gene, not vertically within each sample.

The edgeR package (v. 3.22.5) was used to analyze differentially expressed genes (DEGs) between conditions within each group. DEG analysis was performed with padj (*p*-value adjusted) < 0.05 and a 2-fold change cutoff. The top 30 most significant GO terms from GO enrichment analysis were selected for illustration, or all available terms if the count was below 30. The *X*-axis represents the proportion of differential genes linked to each GO term relative to all differential genes, while the GO terms are represented on the *Y*-axis. Each point’s size indicates the gene count associated with the specific GO term; additionally, the color gradient from red to purple shows enrichment significance values.

### 4.6. Reverse Transcription qPCR

RNA extraction from tissues was performed using an RNA extraction kit (Qiagen), followed by reverse transcription with a RevertAid First Strand cDNA Synthesis Kit (Thermo Fisher Scientific, Waltham, MA, USA) according to the manufacturer’s instructions. qPCR analysis was conducted on the QuantStudio™ 6 system (Thermo Fisher Scientific; Gyeongnam Bio and Anti-aging Core Facility Center) using Luna^®^ qPCR Master Mix (NEB) (Luna, San Jose, CA, USA) following the manufacturer’s guidelines. All primer sequences used are provided in Table S1.

### 4.7. Western Blot Analysis

Tissues were homogenized in radioimmunoprecipitation assay buffer with a bead homogenizer after adding 100× protease inhibitor (Abbkine, Wuhan, China) and 100× phosphatase inhibitor (ChemScene, Monmouth Junction, NJ, USA). The samples were incubated on ice for 20 min, then centrifuged for 10 min at 14,000× *g*. The supernatant was carefully transferred to a new tube after removing the fat, then mixed with an appropriate amount of NuPAGE™ LDS loading buffer (Thermo Fisher Scientific) and subjected to boiling at 95 °C for 10 min. Protein lysates were separated using sodium dodecyl sulfate-polyacrylamide gel electrophoresis and then transferred to polyvinylidene difluoride membranes (Thermo Fisher Scientific) according to the manufacturer’s procedures. Primary and secondary antibodies were diluted at 1:500 to 1:2000 and 1:5000 to 1:10,000, respectively. Protein bands were visualized using ECL chemiluminescence solution (Thermo Fisher) and captured with an iBright™ 1500 Chemi-Doc machine (Thermo Fisher, Waltham, MA, USA). Densitometry analysis was performed using ImageJ gel analysis software (ver. 1.54g). The main figures show representative images, and additional images from at least three independent experiments are presented in the Supplementary Information. Complete, uncropped Western blot images are provided in Supplementary Figure S2.

### 4.8. Lipid Peroxidation Assay

Lipid peroxidation was assessed by quantifying malondialdehyde (MDA) levels in BAT using a Lipid Peroxidation (MDA) Assay Kit (MAK568, Sigma-Aldrich, St. Louis, MO, USA), according to the manufacturer’s instructions. BAT samples were homogenized in MDA lysis buffer at a ratio of 30 μL/mg tissue, and MDA levels were quantified using a standard curve. The results are expressed as nmol MDA per mg tissue (*n* = 8 per group).

### 4.9. Statistical Analysis

Data visualization and statistical analysis were performed using GraphPad Prism software (ver. 8.0.2.). All in vivo experiments were performed with *n* = 6 per group. Depending on the comparison, statistical significance was evaluated using a two-tailed Student’s *t*-test or analysis of variance. Quantitative results are presented as mean ± standard error of the mean from a minimum of three independent experiments. Statistical significance levels are: * *p* < 0.05, ** *p* < 0.01.

## 5. Conclusions

Our research demonstrates that metformin is a potent modulator of brown adipocyte transcriptome patterns, especially in anti-ferroptotic signaling pathways. The coordinated upregulation of ferroptosis resistance genes is accompanied by the induction of Nrf2 target genes and by modulated inflammatory processes. This coordinated response presents a novel mechanistic framework for understanding the protective effects of metformin. These findings support the increasing recognition of ferroptosis as a treatment target and establish metformin as a promising therapeutic option for age-related metabolic disorders. The identification of specific metabolic pathways offers new insights for developing targeted therapies that leverage metformin’s protective mechanisms while minimizing potential side effects.

## Data Availability

Total FPKM values from RNA-seq data are summarized in Table S2, and the raw data are available upon request.

## Supplementary Materials

The following supporting information can be downloaded at: https://www.mdpi.com/article/10.3390/ijms27177625/s1.

## Abbreviations

AMPK, AMP-activated protein kinase; BAT, brown adipose tissue; GPX, glutathione peroxidase; GTT, glucose tolerance test; HFD, high-fat diet; ITT, insulin tolerance test; MDA, malondialdehyde; Nrf2, nuclear factor erythroid 2-related factor 2.
