# Supplementary material for: Metformin Modulates Ferroptosis-Related and Antioxidant Gene Expression in Brown Adipose Tissue
Source: Int J Mol Sci. 2026 Aug 25;27(17):7625. doi: 10.3390/ijms27177625 (PMC13565991; doi:10.3390/ijms27177625)
Supplement: Supplementary file 1 [file ijms-27-07625-s001.zip › Supplementary_Table_S1.pdf]

Table S1. Primer and sgRNA sequence information

Genes	Forward sequence (5`-3')	Reverse sequence (5`-3')
Tslp	GCA AAT CGA GGA CTG TGA GAG C	TGA GGG CTT CTC TTG TTC TCC G
GDF15	AGC CGA GAG GAC TCG AAC TCA G	GGT TGA CGC GGA GTA GCA GCT
Il1rl1	GGA TTG AGG TTG CTC TGT TCT GG	TCG GGC AGA GTG TGG TGA ACA A
Ngf	GTT TTG CCA AGG ACG CAG CTT TC	GTT CTG CCT GTA CGC CGA TCA A
Csf1	GCC TCC TGT TCT ACA AGT GGA AG	ACT GGC AGT TCC ACC TGT CTG T
Tnfrsf21	CAC CTT CCT CTG GCA CAG TTA C	CCT TCC TCG ATG CCA CTT GGT A
Clcf1	GGA GCA TCA ACT CCG CAG CTT A	CCA CAC TTC CAA GTT GAC CGT G
Inhba	GGA GAT AGA GGA CGA CAT TGG C	ACG CTC CAC TAC TGA CAG GTC A
Ccl2	GCT ACA AGA GGA TCA CCA GCA G	GTC TGG ACC CAT TCC TTC TTG G
Tgfb3	AAG CAG CGC TAC ATA GGT GGC A	GGC TGA AAG GTG TGA CAT GGA C
Gdf6	CAC TAG CTT TGT AGA CAG AGG AC	CCT GGC GAT AAA GCC TTA GCT C
Il17ra	CTG TAT GAC CTG GAG GCT TTC TG	CGA GTA GAC GAT CCA GAC CTT C
Tgfb1	TGA TAC GCC TGA GTG GCT GTC T	CAC AAG AGC AGT GAG CGC TGA A
Tnfrsf12a	GAC CTC GAC AAG TGC ATG GAC T	CGC CAA AAC CAG GAC CAG ACT A
Eda2r	AAG CAG ACT CCT TCT TCC GAG G	CAC CAG TGC AAC AAG TGT GGC T
Il13ra1	TGT TCT TGC CGA CGC TGT CTA C	GTG TAG AAG GTG GAG TTT TGC TC
Ccl7	CAG AAG GAT CAC CAG TAG TCG G	ATA GCC TCC TCG ACC CAC TTC T
Tnfrsf11b	CGG AAA CAG AGA AGC CAC GCA A	CTG TCC ACC AAA ACA CTC AGC C
Nr1h3	ATC GCC TTG CTG AAG ACC TCT G	CTG CTT TGG CAA AGT CTT CCC G
Hmox1	CAC TCT GGA GAT GAC ACC TGA G	GTG TTC CTC TGT CAG CAT CAC C
Acsl4	CCT TTG GCT CAT GTG CTG GAA C	GCC ATA AGT GTG GGT TTC AGT AC
Ftl1	CCT CGA GTT TCA GAA CGA TCG C	CCT GAT TCA GGT TCT TCT CCA TG
Gclc	ACA CCT GGA TGA TGC CAA CGA G	CCT CCA TTG GTC GGA ACT CTA C
Sat1	GAG GAT GGC TTT GGA GAA CAC C	GAT ACA GCA ACT TGC CAA TCC ATG
Slc7a11	CTT TGT TGC CCT CTC CTG CTT C	CAG AGG AGT GTG CTT GTG GAC A
Gclm	TCC TGC TGT GTG ATG CCA CCA G	GCT TCC TGG AAA CTT GCC TCA G
Trp53	TGC TCA CCC TGG CTA AAG TT	AAT GTC TCC TGG CTC AGA GG
Slc3a2	GAG CGT ACT GAA TCC CTA GTC AC	GCT GGT AGA GTC GGA GAA GAT G
Gpx4	CCT CTG CTG CAA GAG CCT CCC	CTT ATC CAG GCA GAC CAT GTG C
Ncoa4	TGC CAT TGG TCT TCA GGC TCC T	CAG GCA TCG CTG AAG AAA CTG C
Fth1	GCC GAG AAA CTG ATG AAG CTG C	GCA CAC TCC ATT GCA TTC AGC C
Keap1	ATC CAG AGA GGA ATG AGT GGC G	TCA ACT GGT CCT GCC CAT CGT A
Nrf2	CAG CAT AGA GCA GGA CAT GGA G	GAA CAG CGG TAG TAT CAG CCA G
Slc11a2	TTG CAG CGA GAC TTG GAG TGG T	GCT GAG CCA ATG ACT TCC TGC A
L32	ATC AGG CAC CAG TCA GAC CGA T	GTT GCT CCC ATA ACC GAT GTT GG
